# 3D Ultrasound-Guided Photoacoustic Imaging to Monitor the Effects of Suboptimal Tyrosine Kinase Inhibitor Therapy in Pancreatic Tumors

**DOI:** 10.3389/fonc.2022.915319

**Published:** 2022-07-07

**Authors:** Abigail Claus, Allison Sweeney, Deeksha M. Sankepalle, Brian Li, Daniel Wong, Marvin Xavierselvan, Srivalleesha Mallidi

**Affiliations:** Department of Biomedical Engineering, Tufts University, Medford, MA, United States

**Keywords:** photoacoustic imaging, pancreatic cancer, neoadjuvant therapy, suboptimal therapy, hypoxia, blood oxygen saturation, treatment prediction, tyrosine kinase inhibitor

## Abstract

Pancreatic cancer is a disease with an incredibly poor survival rate. As only about 20% of patients are eligible for surgical resection, neoadjuvant treatments that can relieve symptoms and shrink tumors for surgical resection become critical. Many forms of treatments rely on increased vulnerability of cancerous cells, but tumors or regions within the tumors that may be hypoxic could be drug resistant. Particularly for neoadjuvant therapies such as the tyrosine kinase inhibitors utilized to shrink tumors, it is critical to monitor changes in vascular function and hypoxia to predict treatment efficacy. Current clinical imaging modalities used to obtain structural and functional information regarding hypoxia or oxygen saturation (StO_2_) do not provide sufficient depth penetration or require the use of exogenous contrast agents. Recently, ultrasound-guided photoacoustic imaging (US-PAI) has garnered significant popularity, as it can noninvasively provide multiparametric information on tumor vasculature and function without the need for contrast agents. Here, we built upon existing literature on US-PAI and demonstrate the importance of changes in StO_2_ values to predict treatment response, particularly tumor growth rate, when the outcomes are suboptimal. Specifically, we image xenograft mouse models of pancreatic adenocarcinoma treated with suboptimal doses of a tyrosine kinase inhibitor cabozantinib. We utilize the US-PAI data to develop a multivariate regression model that demonstrates that a therapy-induced reduction in tumor growth rate can be predicted with 100% positive predictive power and a moderate (58.33%) negative predictive power when a combination of pretreatment tumor volume and changes in StO_2_ values pretreatment and immediately posttreatment was employed. Overall, our study indicates that US-PAI has the potential to provide label-free surrogate imaging biomarkers that can predict tumor growth rate in suboptimal therapy.

## Introduction

Pancreatic cancer is one of the leading causes of death worldwide and accounts for approximately 7% of all cancer deaths, with its rate of incidence increasing steadily since 2000 (1). In the United States alone, it is projected that there will be over 49,000 deaths from pancreatic cancer in the year 2022 (1). Unfortunately, the 5-year survival rate for this disease is only 10%, and at most 20% of the diagnosed patients are deemed eligible for surgical resection (1). Several recent studies indicate that neoadjuvant treatment can play a major role in pancreatic cancer treatment, especially in making previously unresectable tumor candidates for surgical resection and organ function preservation (2, 3). The administration of cytotoxic drugs is hampered by heterogeneous distribution of blood flow, hypoxia, and dense stroma commonly found in pancreatic tumors (4). Numerous treatment techniques are predicated on the increased vulnerability of rapidly multiplying tumor cells, but cells in hypoxic areas have a low proclivity for mitosis and thereby may not be exposed to sufficient chemotherapeutic doses (5).

Angiogenesis induction is regarded as a critical phase in tumor development and is one of the characteristics of malignant growth. Vascular endothelial growth factor (VEGF), a group of proangiogenic signaling molecules, and its receptors VEGFR1, VEGFR2, and VEGFR3 also contribute to tumor growth (6, 7). Hepatocyte growth factor (HGF) is a powerful angiogenic factor that works in tandem with VEGF (8, 9). The proto-oncogene mesenchymal–epithelial transition (MET) encodes the receptor tyrosine kinase c-MET factor, otherwise known as an HGF receptor. It is presently the only receptor identified to have a high binding affinity for HGF (10). Activation of the c-MET signaling pathway is normally regulated to sustain cell equilibrium; however, during carcinogenesis, c-MET signaling can become dysregulated by a variety of mechanisms (11). Elevated c-MET protein expression has been shown in many malignancies and has been found to be a robust predictor of poor survival (12–15). Overexpression of c-MET is observed in pancreatic adenocarcinoma, promoting tumor incidence and growth (13).

Tyrosine kinase inhibitors (TKIs) that target multiple pathways such as the c-MET and VEGF pathways are utilized in pancreatic cancer neoadjuvant treatment (16). For example, cabozantinib (XL-184), an orally available TKI, targets both c-MET and VEGFR2. Blocking both arms of the MET/VEGF axis provides major advantages (17). Cabozantinib inhibits its targets in a powerful and reversible manner, causing disruption of cellular processes involved in angiogenesis. This results in severe alterations in tumor physiology, such as extensive endothelial and tumor cell death, vascular disruption, and increased hypoxia. Cabozantinib’s impact on tumors expressing MET and VEGF has been examined with *in vivo* mouse models (17, 18). It substantially enhanced tumor hypoxia (13-fold) and cell death (2.5-fold) at the 8- and 4-h time points after the first and second dosages, respectively. The number of hypoxic and apoptotic cells continuously increased 85- and 78-fold, respectively, following the third treatment (18). These results reiterate the importance of monitoring early treatment response in cabozantinib treatment, as the structural, vascular, and metabolic heterogeneity of tumors can pose a hurdle for effective therapeutic response, and monitoring these changes early on can guide us in understanding cabozantinib therapeutic efficacy.

Clinical decisions are often chosen based on the lack of progression in tumor volume rather than changes in functional or metabolic properties of the tumor. According to Katz et al. (19), radiographic downstaging is uncommon following neoadjuvant therapy, and RECIST response measured on computed tomography (CT) was not regarded as an acceptable therapeutic objective for patients with borderline resectable pancreatic tumors, since only 12% exhibited some form of radiographic response. The majority of studies on neoadjuvant treatment of pancreatic cancer concentrate on radiographic prediction of resectability or rates of radiographic downstaging rather than attempting to identify possible functional predictors of tumor response (20–22). There is an absence of effective imaging techniques and serological biomarkers for the assessment of tumor response for neoadjuvant chemotherapy in resectable and borderline resectable pancreatic cancer (23). Gauging the tumor microenvironment (TME) heterogeneities, particularly vasculature and function, i.e., tumor oxygenation status, at high resolution is needed to both evaluate treatment response and predict recurrence at an early phase during treatment (24). Currently, macroscopic imaging modalities such as positron emission tomography (PET) scans and magnetic resonance imaging (MRI) are sparingly used clinically for pancreatic cancer to gauge therapy-induced changes in the TME during chemo or radiotherapy (25) and are yet to establish themselves as tools in monitoring therapy response in pancreatic cancer. In fact, a review by Granata et al. (26) mentions that no study has reported the usage of blood oxygen level-dependent (BOLD) MRI in pancreatic cancer. Furthermore, these modalities utilize exogenous contrast agents whose pharmacokinetics in the body limit the frequency of imaging (often several weeks) leading to the loss of crucial information on TME modulation due to therapy at early time points posttreatment. Hence, there is a dire need for a noninvasive, non-ionizing, label-free quantitative imaging modality that can longitudinally monitor and tease out dynamic changes in the TME at early time points post-therapy. Photoacoustic imaging (PAI) is a non-ionizing imaging modality that involves a short nanosecond laser pulse irradiating a biologic tissue sample to visualize optically absorbing internal structures in the tissue (27). This technique can acquire images of tumor vasculature [contrast provided by hemoglobin (Hb)] and oxygen saturation (StO_2_) without the need for exogenous contrast. StO_2_ is the ratio of hemoglobin binding sites that are occupied by oxygen and is calculated by dividing oxygenated hemoglobin (HbO_2_) by the total hemoglobin (HbT). Both oxygenated and deoxygenated Hb exhibit a molar extinction coefficient that is at least one order of magnitude greater than other common chromophores such as lipids at wavelengths between 650 and 900 nm, and spectral unmixing of photoacoustic measurements made with at least two wavelengths within this wavelength range can quantitatively approximate StO_2_ values (28). PAI has the potential to be a scalable tool to enable patient stratification and monitor therapy response (29). Given the wide utility of ultrasound (US) in clinical imaging of pancreatic tumors, we believe that ultrasound-guided photoacoustic imaging (US-PAI) can simultaneously provide structural (tumor volume and shape) and functional (vascular StO_2_) information of pancreatic tumors (28, 30, 31).

Several studies demonstrated the utility of PAI for vascular characterization of preclinical tumor models and monitoring therapy response. For example, Hacker et al. (32) used PAI to explore the relationship between HbT and StO_2_ photoacoustic biomarkers and the underlying biochemical blood parameters in a species-specific manner. In a study by Keša et al. (33), quantitative *in vivo* monitoring of hypoxia and vascularization of mantle cell lymphoma using US-PAI was performed. They analyzed levels of oxygen and vascularization in immunocompromised mice to not only show the reproducibility of US-PAI data but also qualify US-PAI as a valuable noninvasive imaging modality. In a study by Rich et al. (34) and Tomaszewski et al. (35), PAI has been shown to be a useful tool in revealing tumor hemodynamics in patient-derived xenograft models of head and neck squamous cell carcinoma in murine models. These studies demonstrated that significant changes in tumor hemodynamics correlated well with treatment outcomes in response to radiation therapy. They found that PAI-based changes in StO_2_ were detected at early time points even before changes in tumor volume were observed. However, this study did not choose to examine pretreatment conditions (i.e., volume or baseline StO_2_). A recent study by Liapis et al. (36) longitudinally examined tumor hemodynamics in two types of breast cancer xenografts focusing particularly on HbT and StO_2_ changes with the administration of bevacizumab, a drug that targets circulating VEGF and prevents it from attaching to its cell membrane receptors. They observed a sharp drop in tumor StO_2_ and HbT concentration shortly after initiation of treatment that was then restored back to pretreatment levels. Liapis et al. (36) highlight the importance of data collection immediately following the administration of therapeutic agents. Similarly, Hysi et al. (37) examined changes in tumor oxygenation in murine breast cancer models through frequency analysis of photoacoustic radiofrequency signals and StO_2_ *in vivo* throughout the administration of thermosensitive liposomes encapsulated with doxorubicin. They correlated spectral slope with treatment-induced hemorrhaging to differentiate treatment responders from non-responders. The observed results demonstrate the potential of US-PAI to not only monitor tumor hemodynamics but also quantify treatment-induced functional changes. None of the studies discussed the combined effects of pretreatment tumor volume, baseline StO_2_, and their impact on tumor response.

The clinical applications of PAI are also rapidly expanding, especially in the diagnosis and characterization of various malignancies. The work of Nandy et al. (38) displayed the feasibility of using functional parameters gathered by pulse-echo US combined with photoacoustic tomography (PAT) to diagnose ovarian cancer. Their research found that relative HbT concentrations were on average 1.9-fold greater in invasive epithelial ovarian cancers than healthy tissue and that StO_2_ was 8.2% higher in healthy ovaries than in invasive tumors (38). In another study, Kim et al. (39) utilized multispectral PAI to stratify thyroid nodules. Using parameters gathered from the photoacoustic spectral gradient, relative StO_2_ levels, and the skew angle of StO_2_ distributions, Kim et al. (39) were able to diagnose papillary thyroid cancer with a specificity of 93% and a sensitivity of 83%. The potential of PAI in assessing cancer response to neoadjuvant chemotherapy was also demonstrated by Lin et al. (40), where images of the breast were taken at three time points (once before, during, and after receiving chemotherapy), while the unaffected breast acted as a control. They observed noticeable decreases in the relative vascular density, entropy, and anisotropy of tumors treated with neoadjuvant therapy (40). While these studies demonstrate the clinical value of PAI, we further display the potential of PAI in oncology by using multiple hemodynamic parameters gathered during the chemotherapeutic regimen to detect non-responsiveness in suboptimal therapeutic regimens and especially predict tumor growth rates.

Previously, our group demonstrated that volumetric PAI can successfully predict the treatment response of vascular targeted photodynamic therapy and changes in StO_2_ levels within 6 and 24 h posttreatment in order to reliably predict recurrence (41). The above-listed studies, including our previous work, showcase the potential of US-PAI in monitoring changes in StO_2_ post-therapy where the therapies were specifically targeting the vasculature and caused almost complete remission of the tumors. In addition, absolute values observed posttreatment were considered; however, relative changes in StO_2_ pretreatment and posttreatment were not extensively explored in suboptimal therapies. This study built upon all the above-stated literature to monitor the response to treatment by quantifying pretreatment tumor conditions and evaluating utility in treatment prediction when combined with posttreatment tumor conditions. Additionally, we correlated tumor growth measurements against the vascular functional data acquired with US-PAI. Overall, we showcase the utility of label-free multiparametric US-PAI in monitoring pretreatment and posttreatment changes in StO_2_ and in predicting tumor growth rates in suboptimal TKI therapy in subcutaneous pancreatic tumor models.

## Materials and Methods

### Cell Line Preparation

AsPC-1 (pancreatic adenocarcinoma) cells obtained from the American Type Culture Collection were cultured in RPMI 1640 medium. The medium was supplemented with 10% fetal bovine serum and 1% penicillin-streptomycin (100 U/ml). Cells were passaged 1–2 times per week and maintained in an incubator in 5% CO_2_ at a temperature of 37°C.

### Animal Protocol and Cell Implantation

The Institutional Animal Care and Use Committee (IACUC) of Tufts University authorized all animal experiments conducted in this study. Male homozygous Foxn1^nu^ nude mice (6–8 weeks) were sedated with 1% isoflurane USP and subcutaneously injected with 5 million AsPC-1 cells on day 0 (abbreviated as “D0” throughout the article. Respective days follow similar convention; for example, Day 5 post-implantation is abbreviated as D5). The cells (passage numbers 15–19) were delivered in 100 µl of Matrigel (50 µl of Matrigel + 50 µl of phosphate buffered saline) using a 1-ml insulin syringe (29-gauge).

### Drug Administration

On D11, mice were split into two randomized groups of control (no-treatment) mice and cabozantinib-treated mice. Cabozantinib (Cat. No.: HY-13016, MedChemExpress) solution was made with 30% polypropylene glycol and 5% Tween-80, and 65% D5W (dextrose 5% water) and administered daily at 1 mg/kg (n = 5), 10 mg/kg (n = 4), 30 mg/kg (n = 5), or 100 mg/kg (n = 8) *via* oral gavage for 2 weeks, except on the weekends. A total of 29 mice were used in the study, where 15 mice (no-treatment and 100-mg/kg group) were used initially to gauge the significance of the predictors, and the multivariable regression models were developed using data from all of the 29 mice in the study.

### Ultrasound-Guided Photoacoustic Imaging Acquisition

**Figure 1:**
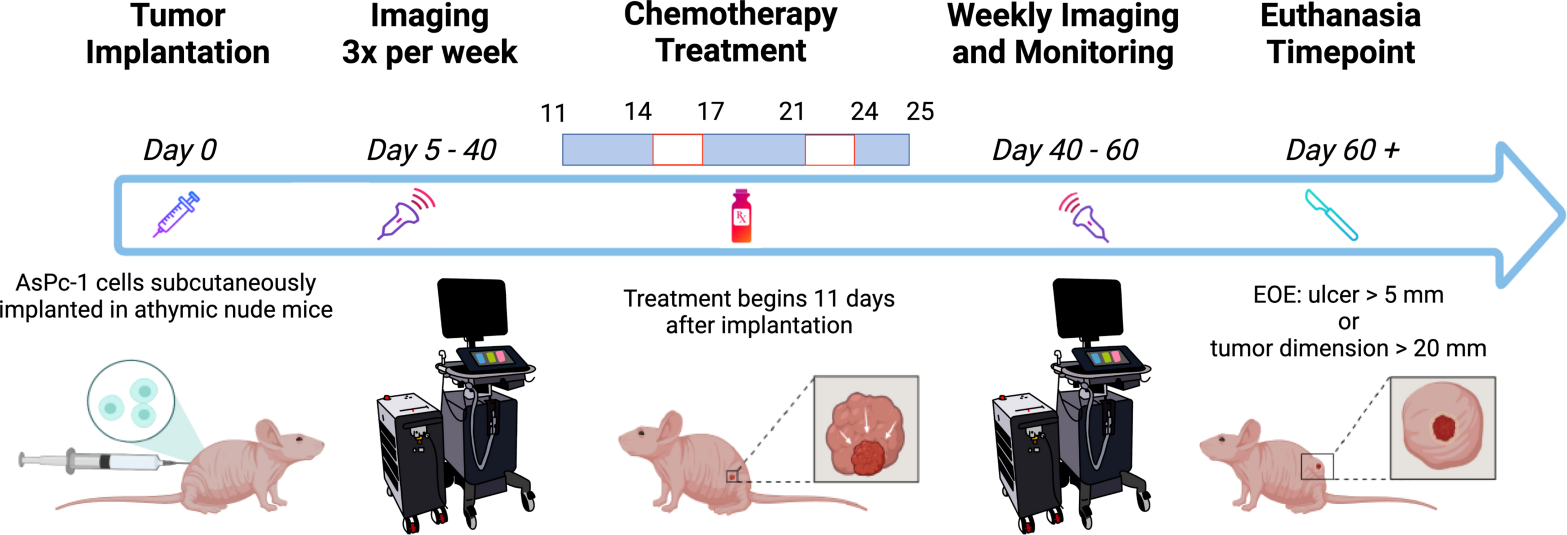
Schematic of the experiment timeline. The mice were imaged frequently before and during chemotherapy treatment. Weekly imaging ensued 40 days after tumor implantation through the end of the experiment (EOE). Created with Biorender.com.

Image acquisition was performed with the Vevo LAZR-X US-PAI system (FUJIFILM VisualSonics, Inc.) that was equipped with a Nd : YAG nanosecond pulsed laser and optical parametric oscillator (OPO) operating at 20-Hz pulse repetition frequency. The laser pulse duration was 4–6 ns and was tunable between 680 and 970 nm, providing a peak energy of 45 ± 5 mJ. Linear array transducer MX250S (15-30 MHz) operating at a center frequency of 21 MHz was used to obtain ultrasound and photoacoustic images at 750 and 850 nm, wavelengths currently available with the “Oxy-Hemo” mode on the Vevo LAZR-X system. The two wavelengths were chosen, as they straddle an isosbestic point of oxygenated and deoxygenated hemoglobin, facilitating the calculation of HbT and (StO_2_). The ultrasound and PAI gain were set to 22 and 45 dB, respectively, for all imaging sessions in the study. Persistence and acquisition were set to “Maximum” and “Oxy-Hemo” modes, respectively, for obtaining the HbT and StO_2_ of the tumors. Two types of oxygen saturation values were measured, as follows: 1) StO_2_ average: an average of StO_2_ values of the pixels both with and without non-zero photoacoustic signal within the region of interest (ROI) and 2) StO_2_ total: an average of all StO_2_ values of all the pixels within the ROI. StO_2_ total values are lower than StO_2_ average because they considered all regions within the volumetric scan. HbT average and HbT total values are acquired in a similar fashion. Prior to image acquisition, mice were anesthetized with 2% isoflurane. Tumor dimensions [length (L), width (W), and height (H)] with digital calipers and photographs were obtained. Tumor volume was calculated using the formula $\frac{\left(L\cdot W\cdot H\cdot \pi \right)}{6}$ . The anesthetized mice were placed on a heated imaging table maintained at a temperature of approximately 37°C and connected to the ECG leads to monitor the heart rate throughout data acquisition. Optically clear ultrasound gel (Aquasonic 100 Ultrasound Transmission Gel, Parker Laboratories, Inc.) was applied to the tumor and surrounding region to allow for effective acoustic transmission between the transducer and the tissue. Each frame in the acquisition is composed of approximately 20 images acquired at 750/850-nm wavelengths. These images, together, produced a cross-sectional US-PAI B-scan image of the tumor. A three-dimensional (3D) scan of the tumor was performed with 0.15 mm step size. As the lateral resolution of the transducer array was approximately 300 µm, a step size of 150 µm was chosen to satisfy Nyquist criterion. After image acquisition, the mice were returned to clean cages for safe recovery post-imaging. The mice were imaged three times per week beginning on D5 post-tumor implantation and continued through approximately D40 (**Figure 1**). After the first day of treatment (D11), the mice were imaged 24 h after and then frequently afterward (**Figure 1**). Mice were euthanized if the tumor length reached 20 mm in any direction or if the tumor developed ulcerations of length 5 mm or greater.

To evaluate the impact on StO_2_ measurements due to depth-dependent optical attenuation of light, we conducted a phantom experiment in which oxygenated (100% StO_2_) and deoxygenated (0% StO_2_) blood inside a 2-mm diameter polyethylene tube was imaged (with the same settings as tumor images) at depths ranging from 5 to 18 mm from the transducer. The tube was placed in a tank filled with water (no optical scattering) and in 0.5% intralipid solution to mimic tissue scattering (42, 43). Three separate experiments were conducted. The blood solution was prepared with 2.5 mM bovine hemoglobin (Sigma Aldrich) in phosphate buffer solution, and the partial pressure of oxygen (pO_2_) of the solution was measured consistently throughout the imaging session using an Oxylite electrode sensor (Oxford Optronix).

### Image and Data Processing

**Figure 2:**
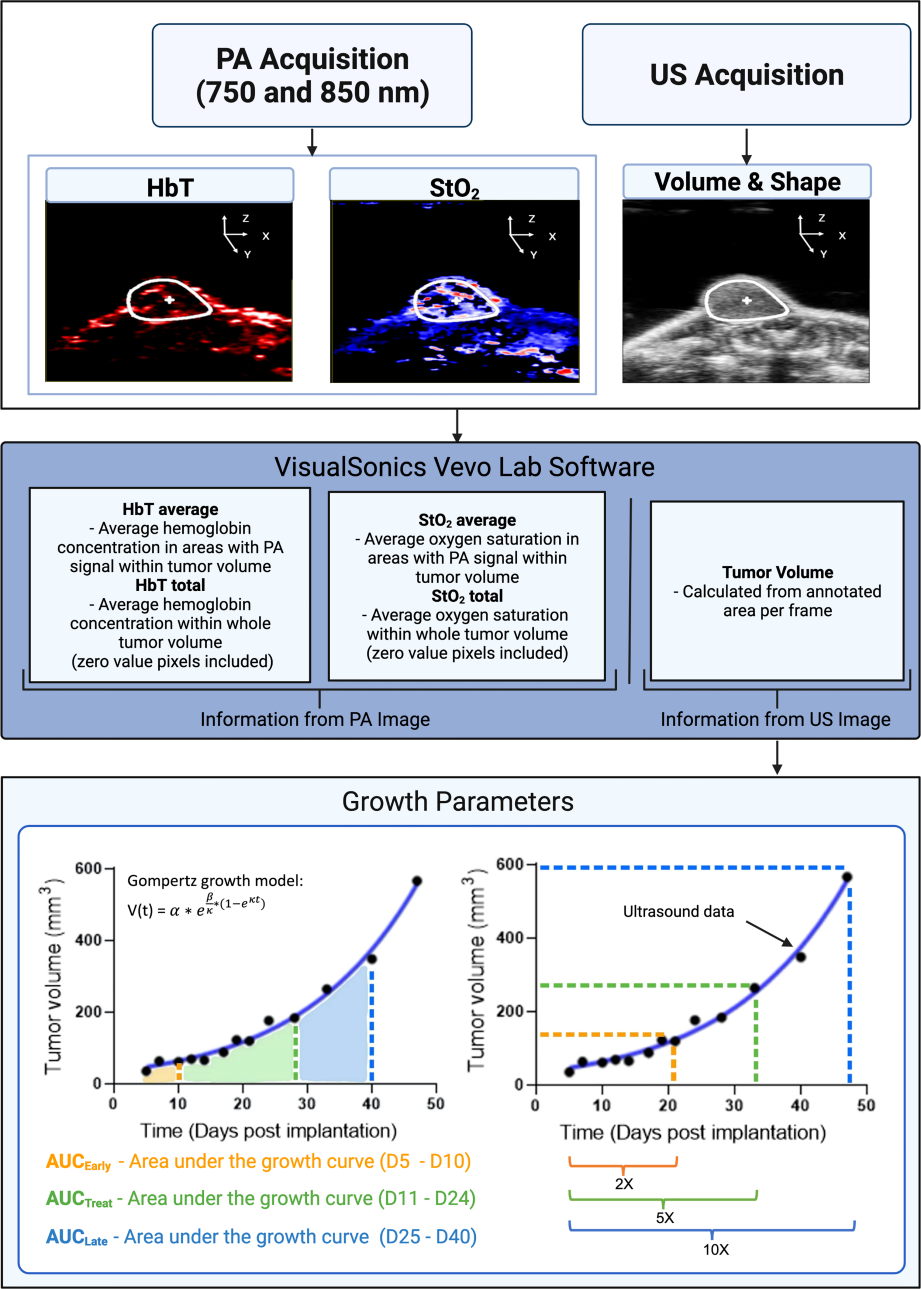
A flowchart depicting the steps involved in collecting and analyzing relevant data in our experiment. Inputs consist of StO_2_, HbT (from photoacoustic images), and tumor volume (extracted from ultrasound images). Tumor volume gathered from ultrasound images was plotted for various days post-implantation. The data were fitted with the Gompertz growth model. Shown in the bottom left schematic representation is the area under the curve (AUC) for different time periods during the study. AUC_Early_ (orange shaded area) is area under the volume vs. time plot during the pretreatment days (D5–D10). AUC_Treat_ (green shaded region) represented the AUC for time period D11–D26 when the mice were treated with cabozantinib. AUC_Late_ (blue shaded region) is the AUC for time period D26–D40. AUC parameters units are mm^3^ * day. The bottom right image is a schematic representation of the tumor volume vs. days post-implantation curve utilized to determine the 2×, 5×, and 10× growth parameters. 2× is the number of days it takes for a tumor’s pretreatment (D5) volume to double in size. Green (5×) and blue (10×) show the number of days it takes for pretreatment volume to increase by factors of 5 and 10, respectively. X-fold increase parameters are measured in terms of days post-implantation. PA and US represent photoacoustic and ultrasound, respectively. Created with Biorender.com.

The VevoLab software (VisualSonics, Toronto, ON, Canada) was used to render 3D images of the tumors at various time points to qualitatively compare changes in StO_2_ values. Tumor regions were also segmented, and custom written MATLAB scripts were developed to extract the volume, StO_2_ and HbT values from each imaging data set obtained from VevoLab. Tumor volume obtained on various days was fit using the $\alpha *e^{\left(\frac{\beta }{K})*(1-e^{-K*t}\right)}$ where α is the initial tumor volume, β is the initial specific growth rate, κ is the retardation parameter, i.e., it is the rate of exponential decay of initial specific growth rate, and t is time (44). The Gompertz function is widely used and has been shown to provide a good fit for numerous tumor models undergoing therapy, where the tumor growth is slowest at the end of a time period (44). Specifically, here we are interested to evaluate if pretreatment tumor growth influences the tumor’s ability to respond to cabozantinib treatment. The area under the tumor growth curve for D5, D7, and D10 is termed AUC_Early_, for D10–D21 during the treatment is termed AUC_Treat_, and for D21–D40 is termed AUC_Late_ as depicted in **Figure 2**. The exponential fits of the tumor volume data were also used to calculate the time taken for the tumor to double in size, increase 5-fold and 10-fold in volume (**Figure 2**).

### Statistical Analysis

We used GraphPad Prism (La Jolla, CA, USA) to complete the statistical analyses including the correlation matrix and multiple logistic regression analysis. Multiple linear regression analysis with 5-fold cross-validation method and repeated random subsampling validation method (100 repeats) were performed on MATLAB. Multiple unpaired t-tests were used to compare the control and cabozantinib groups on each day post-implantation, and a p-value <0.05 considered statistically significant unless specifically stated.

## Results and Discussion

### Effect of Suboptimal Cabozantinib Treatment on Tumor Growth Rate

**Figure 3:**
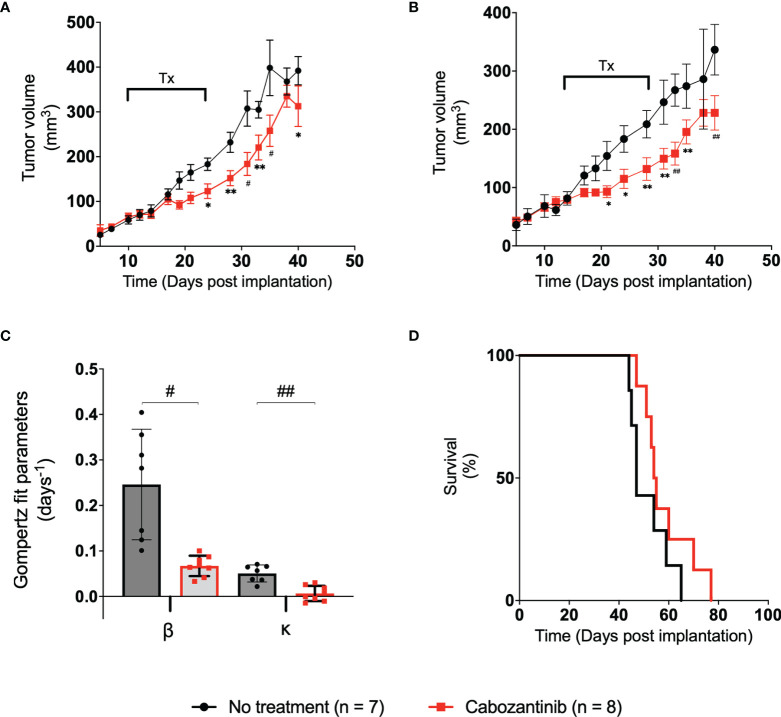
**(A)** Plot of tumor volume obtained from ultrasound images vs. the time post-tumor implantation. The treatment days are indicated with a bar labeled “Tx.” **(B)** Plot of tumor volume measured with digital calipers vs. days post-tumor implantation. Error bars represent the SEM for each of the groups on a particular day. **(C)** Bar plot of the spread of each group’s Gompertz growth rates. Error bars represent SEM, where n = 8 for each group. **(D)** Kaplan–Meier survival curve of the two groups over the period of the study. Median survival time for control and treated groups was 47.5 and 54.5 days, respectively. * p < 0.05, ** p < 0.01, # p < 0.001, ## p < 0.0001.

The tumor volumes of mice in the control group and the 100-mg/kg cabozantinib-treated group are shown in **Figure 3** black and red lines, respectively. The control (no-treatment) tumors grew at a faster rate than the tumors that received cabozantinib as expected. Performing multiple two-tailed unpaired t-tests between the two groups, volumes on D21, D24, D28, D31, D33, D35, and D40 show statistical significance (**Figure 3A**) (**Table S1**). We observed that the tumor volume measured with digital calipers also demonstrated statistically significant differences between the treated and the control group on the same days above (**Figure 3B**) and (**Table S2**). The B-scan images of the tumors (center frame) from representative mice in the two groups are provided in **Figure S4**. Qualitative change in tumor size cannot be discerned on the 2D images, and the effect of the suboptimal treatment is only apparent when the entire tumor volume is considered. Gompertz growth parameters β and κ representing initial growth rate and retardation parameter are displayed in **Figure 3C**. Clearly, a 4- and 8-fold decrease in the respective growth rate parameters can be observed between the two groups. A two-tailed unpaired t-test demonstrated that there is a strong statistically significant difference between the tumor growth rates (p < 0.006) of the two groups due to the effect of cabozantinib (**Figure 3C**).

Examining the AUCs of the tumor volume vs. treatment days curve (schematic representation in **Figure 2**) of the control and treated groups during the experiment, no statistical significance was observed between the AUC_Early_ and AUC_Treat_ days. However, AUC_Late_ values between the groups showed statistically significant differences through a two-tailed unpaired t-test (p = 0.026) (**Figure S1**), indicating that the administration of the drug, even in suboptimal doses, has had a measurable effect at later days posttreatment. No statistically significant difference in mouse weight was observed in this study (**Figure S2**). The effect of cabozantinib is also clearly demonstrated through the Kaplan–Meier survival curves. Median survival time in mice was 47.5 and 54.5 days for control and treated groups, respectively (**Figure 3D**). Approximately around D40 is where the survival curve of each group diverges with the control group at risk of death sooner compared to those tumors that received cabozantinib. Clearly, cabozantinib reduced the tumor volume; however, we do not observe complete tumor remission due to suboptimal treatment.

### Oxygen Saturation Values at Early Time Points Are Significant Biomarkers of Treatment Efficacy

To determine the variability in the StO_2_ and HbT value measurements due to laser energy fluctuations, a repeatability experiment was conducted in which the same mouse (n = 3 mice) was imaged three separate times 2 h apart. To approximately coregister the same frames from the separate time points, the center frame of each tumor was found and 20 frames before and after the center frame were compared to the corresponding frames at the other time points. The variance of the StO_2_ average and StO_2_ total parameters was found to be 4.8% ± 1.78% and 7.40% ± 2.27%, respectively, while the average variance of HbT average and HbT total was found to be 3,006 ± 1,106 (au) and 3.96 × 10^7^ ± 1.96 × 10^7^ (au), respectively (**Table S3**). These results enable us to attribute significant differences in StO_2_ values between the treated and control groups to treatment effects if they are greater than the variance mentioned above.

**Figure 4:**
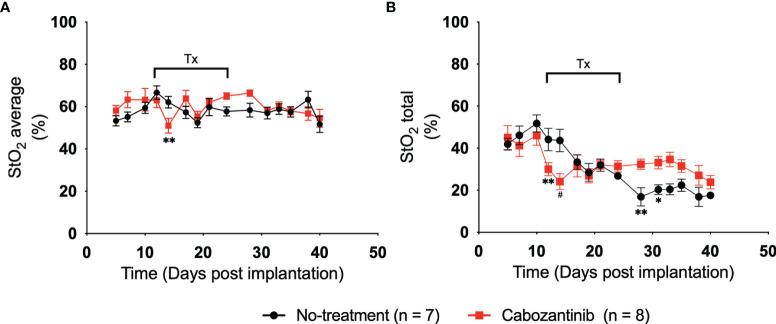
**(A)** Plot of 3D average StO_2_ in the tumors on different days post-implantation. Three days after treatment initiation, there is a statistically relevant difference between treated and non-treated tumors with a p-value of 0.0091. Error bars represent standard error of the mean (SEM) for each day post-implantation. **(B)** Plot of 3D total StO_2_ on different days post-implantation. By performing multiple two-tailed unpaired t-tests, both 24 and 72 h posttreatment have significant differences between the values, with p-values of 0.0083 and 0.0003, respectively. Error bars represent SEM. * p < 0.05, ** p < 0.01, # p < 0.001.

**Figure 4** shows the average and total StO_2_ change in the tumor over time for the control (black line) and cabozantinib group (red line). Multiple two-tailed unpaired t-tests were performed between the two groups. As expected, pretreatment StO_2_ values (both average and total) showed no significant differences between the groups on D5, D7, and D10 post-implantation. Therefore, any differences seen posttreatment can be attributed to the administration of the drug. Within 72 h post-drug administration, statistically significant differences in StO_2_ average were observed on D14 post-implantation (p = 0.009, **Table S4**). These differences were significantly larger than the variance observed in the repeatability experiment. The StO_2_ total values of the groups were different on both D12 and D14 post-implantation (p = 0.008 and p = 0.0003, respectively, **Table S5**). The changes in StO_2_ values do not correspond to changes in HbT values, i.e., no significant differences between the groups were observed in HbT posttreatment (**Figure S3**; **Tables S6, S7**). Using a variety of immunofluorescence assays and histology biomarkers, cabozantinib was shown to inhibit MET and VEGFR phosphorylation and disrupt tumor vasculature in digitally captured histology images (18, 45, 46). As a result, previously present functional blood vessels had been cut off, leading to further hypoxia and an overall decrease in oxygenation of the tumor posttreatment. Furthermore, mimicking a clinical scenario, cabozantinib was not administered on the weekends during the study. During this downtime, the StO_2_ values recovered back to their pretreatment stage, and beyond D17, no statistical significance for the StO_2_ average values was observed. StO_2_ total, on the other hand, had statistical significance on D28 and D31 posttreatment (p = 0.004 and p = 0.029, respectively). Given that the tumors were significantly larger at these time points and no depth-dependent fluence compensation was performed on our StO_2_ measurements, the differences observed at D25 and beyond might not accurately represent the tumor oxygenation status.

**Figure 5:**
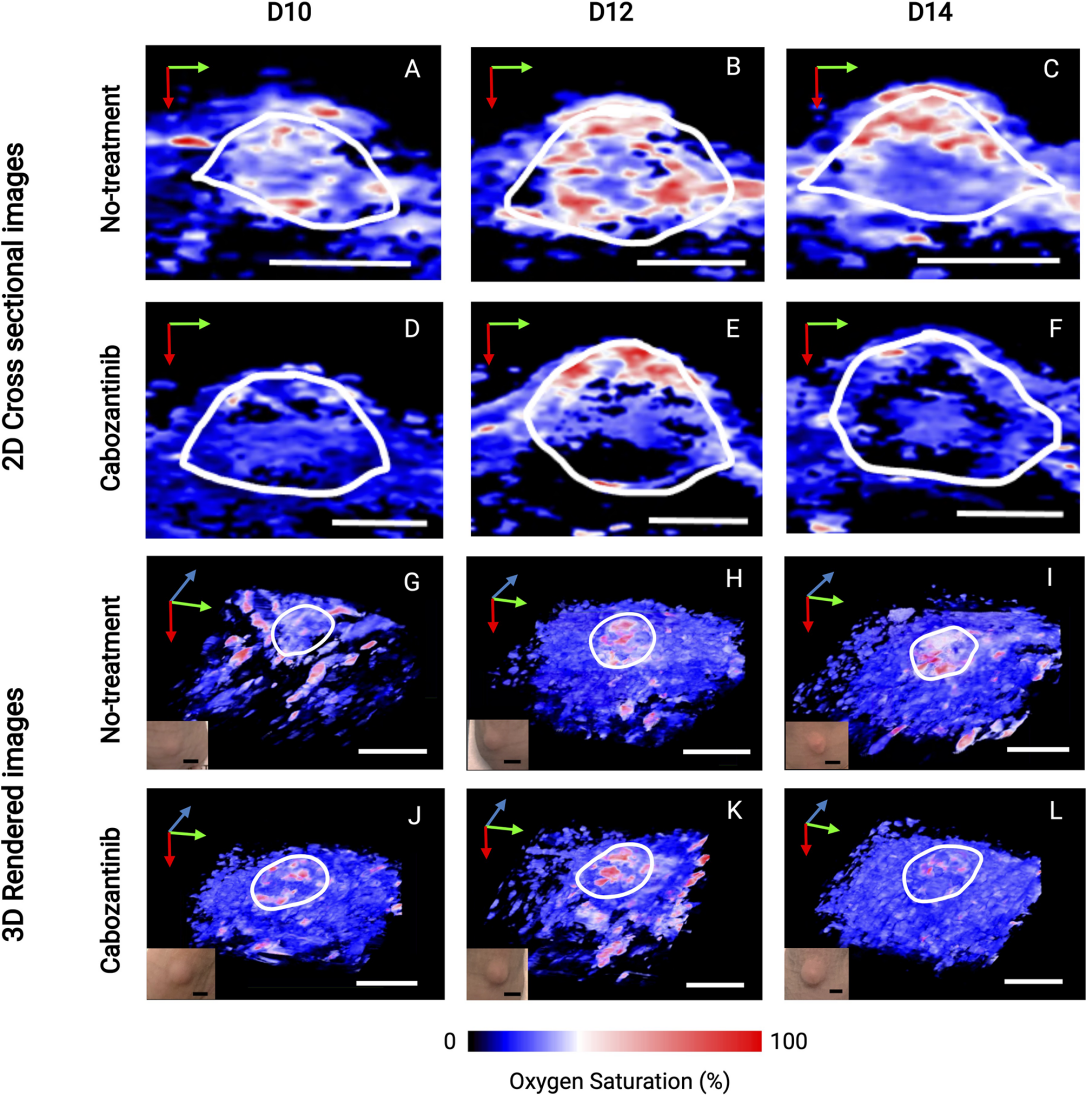
2D cross-sectional photoacoustic images **(A–F)** and corresponding 3D rendered images **(G–L)** of tumor regions from the day before the first administration of treatment (D10) immediately through the first 72 h after the start of treatment (D14). Post-administration of cabozantinib, we observe a decrease in StO_2_ from D12 to D14, while the Control (no-treatment) group had relatively similar StO_2_. Insets shown in the lower left corner of 3D images are photographs of tumors taken immediately before the corresponding PAI acquisition. The tumor volume change at these time points is not statistically significant, indicated also by the no obvious changes seen in the photographs of the tumors. In upper 2D cross-sectional images, scale bars = 2 mm. In lower 3D rendered images, both black and white, scale bars = 5 mm. Green, blue, and red arrows indicate the x-, y-, and z-direction, respectively.

**Figure 5** depicts the 2D US-PAI B-scans and 3D images of representative tumors (outlined in white ROI) at immediately pretreatment and posttreatment time points (D10, D12, and D14) along with respective photographs of the tumors. These images serve as qualitative benchmarks to confirm the quantitative trends observed in **Figure 4**. The images clearly depict that for the treated tumor (**Figures 5D–F, J–L**), the first initial dose decreases the oxygenation in the tumor region. The tumor StO_2_ then continues to decrease as the regimen continues, behavior consistent with how TKI therapy has been demonstrated to work in previous studies (18). Tumors that were not treated (**Figures 5A–C, G–I**) did not exhibit a significant change in their oxygenation status during this time. Moving forward, being able to identify and characterize this type of behavior early during the treatment regimen will be critical to predict response and plan subsequent therapies.

### Changes in Oxygen Saturation Can Predict the Treatment Response

**Figure 6:**
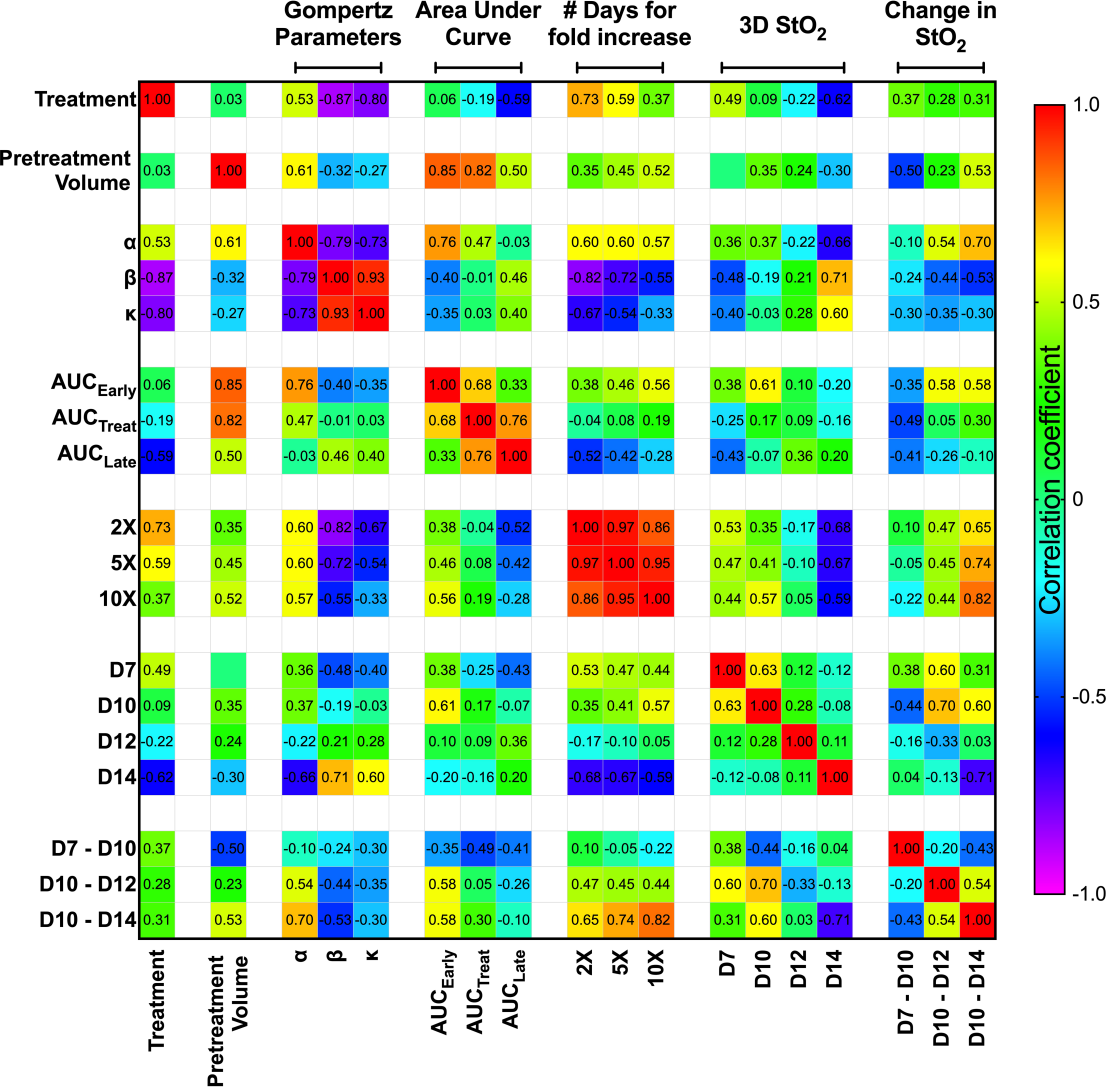
Spearman correlation matrix comparing the tumor growth characteristics and tumor StO_2_ parameters. Tumor growth parameters include pretreatment volume, Gompertz function parameters (α, β, and κ), area under the growth curve obtained pretreatment (AUC_Early_), during treatment (AUC_Treat_), posttreatment (AUC_Late_), and time taken for the tumors to reach twice (2×), 5 times (5×), and 10 times (10×) their pretreatment volume. The StO_2_ parameters include the 3D StO_2_ average values on Days 7, 10, 12, and 14 post-implantation (D7, D10, D12, and D14, respectively) and parameters describing the change in StO_2_ from Day 7 to Day 10 (D7–D10), Day 10 to Day 12 (D10–D12), and Day 10 to Day 14 (D10–D14). Color gradient assists in identifying the most prominent parameter relationships, with red representing a positive correlation coefficient and violet representing a negative correlation coefficient.

To investigate whether any StO_2_ measurements or change in StO_2_ measurements due to therapy could predict a suboptimal treatment response or correlate with various growth parameters, we performed a correlation analysis on multiple parameters as shown in **Figure 6**. The “treatment” parameter had a value of “0” for control mice and “1” for mice treated with cabozantinib. The matrix is organized by growth rate values and tumor volume measurements, then average StO_2_ values at early time points and their relative differences. Specifically, pretreatment volume on D10, Gompertz function parameters (α, β, and κ), area under the growth curve obtained at pretreatment (AUC_Early_), during treatment (AUC_Treat_), posttreatment (AUC_Late_), and time taken for the tumors to reach twice (2×), 5 times (5×), and 10 times (10×) their pretreatment volume (parameters represented in **Figure 2**) were correlated with StO_2_ values. The abbreviations D7, D10, D12, and D14 in the matrix represent the StO_2_ average values on Days 7, 10, 12, and 14 post-tumor implantation, respectively. Positive correlations are represented in red to green hues, while negative correlations are represented in blue to purple hues, and the correlation coefficient is displayed on the matrix for each parameter pair. Although StO_2_ total and average values were both analyzed as shown in **Figure 4**, StO_2_ total was not considered in the correlation matrix, as it could be over or underestimated based on differences in tumor volume. The StO_2_ total calculations include zero-pixel values that could lead to increased error as the tumor size or hypoxia increases, in addition to effect on StO_2_ measurements in larger tumors due to light penetration. Hence, for the treatment prediction model, we used StO_2_ average values and time points early in the treatment regimen where tumor depth differences between D7 and D10 or Day 10 and Day 14 are on the order of ~600 μm. In addition, the correlation coefficients between StO_2_ total and the growth parameters were poor compared to StO_2_ average (**Table S8**) and hence were not used in further analysis.

We used Spearman correlation in our analysis contrary to the standard Pearson correlation values, as we wanted to evaluate the monotonic relationship between the variables, where the variables tend to change together but not necessarily at a constant rate. Using the ranked system in Spearman correlation analysis is more effective for our dataset, as it allows for the evaluation of nonlinear trends and correlation of data from various observations or analyses. The correlation matrix format allowed us to identify the parameter relationships that are most revealing about responsiveness to treatment at the earliest time. As expected, treatment condition highly correlated with Gompertz growth parameters β (r^2^ = -0.87, p = 3.12e-4) and κ (r^2^ = -0.80, p = 0.001). The treatment condition also has a high negative correlation with number of days for increase in volume [r^2^ = 0.73 (p = 0.005), r^2^ = 0.59 (p = 0.025) for 2× and 5×, respectively]. These results agree well with previous studies that have shown that cabozantinib therapy decreases the tumor growth rate (18). Gompertz α parameter, which represents the initial value of the growth curve fit, correlated with pretreatment volume (r^2^ = 0.614, p = 0.02) and AUC_Early_ (r^2^ = 0.76, p = 0.001) as expected, as they theoretically represent the same parameter. It also correlates well with the number of days taken for tumor volume to increase, as larger tumors take less time to reach a certain volume [r^2^ = 0.6 (p = 0.020), r^2^ = 0.6 (p = 0.020), r^2^ = 0.57 (p = 0.028) for 2×, 5×, and 10×, respectively]. Despite AUC_Early_ and AUC_Treat_ both displaying strong correlations with several growth parameters, AUC_Late_ did not display a high correlation with any of the growth parameters. This could be due to several factors, as larger late-stage tumors are susceptible to stalled growth, necrosis, and hemorrhage formation, which can impact the correlation with other parameters. Overall, given the high correlation of the Gompertz growth parameters with the treatment condition, we isolated these relationships and performed further analysis to predict these parameters utilizing the StO_2_ values.

The StO_2_ values on D7, D10, D12, and D14 were chosen to represent the immediate pretreatment and posttreatment values. The Spearman correlation coefficient between the treatment condition and 3D StO_2_ on D14 was -0.62 (p = 0.021). Furthermore, **Figure 3** clearly shows that the differences in StO_2_ between control and cabozantinib-treated groups are statistically significant on D14. The D14 StO_2_ values also have a high correlation with the Gompertz growth parameters [r^2^ = -0.66 (p = 0.009), r^2^ = 0.71 (p = 0.004), r^2^ = 0.60 (p = 0.020) for α, β, and κ, respectively] and the number of days for fold increase [r^2^ = -0.68 (p = 0.001), r^2^ = -0.67 (p = 0.008), r^2^ = -0.59 (p = 0.022) for 2×, 5×, and 10×, respectively].

Pretreatment tumor volume was a significant predictor for tumor growth rate and treatment response in several clinical studies, i.e., larger tumors are less likely to respond to therapy (47, 48). This is also displayed in our results as AUC_Treat_ negatively correlated with pretreatment volume (r^2^ = -0.82, p-value = 0.0003). This indicates that AUC_Treat_ will be larger for smaller tumors and *vice versa*. However, we do not observe a significant correlation between the size of the tumor (pretreatment volume) and StO_2_ values on various days. This observation is supported by the fact that tumors are heterogeneous by nature. Previously, Mallidi et al. (41) demonstrated that the change in StO_2_ post-therapy is predictive of photodynamic therapy response where the therapy specifically targeted blood vessels and 70% of the tumors had complete remission. Pre-therapy changes in StO_2_ values were not correlated to treatment response. Furthermore, a study by Ueda et al. (49) demonstrated that baseline pretreatment StO_2_ can predict a pathologic complete response in breast cancer patients. Here, we test our hypothesis that the change in StO_2_ values immediately pretreatment and posttreatment can predict tumor growth rate parameters, as complete remission rarely occurs in suboptimal therapy.

**Figure 7:**
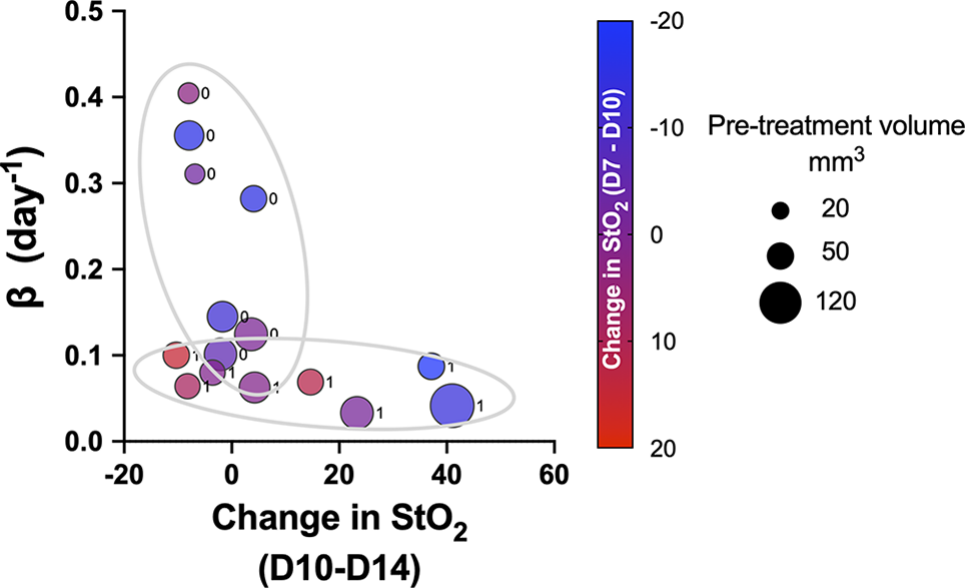
Scatter bubble plot of change in StO_2_ values between D10 and D14 vs. tumor growth rate *β* values. The change in StO_2_ from D7 to D10 is indicated by the color bar where a decrease in oxygenation is represented by red, while an increase in oxygenation is represented by blue. The size of the bubbles is representative of the pretreatment volume of the tumors on D10. The label 0 indicates tumors in the control group, while 1 corresponds to treated.

Pretreatment change in StO_2_ values (D7–D10) had a weak negative correlation with pretreatment tumor volume (r^2^ = -0.504, p-value = 0.037). This indicates that rate of tumor growth and vessel development can be heterogeneous, but generally larger tumors had higher D10 StO_2_ values than at D7. On the other hand, in smaller tumors, StO_2_ values had minimal changes between D7 and D10. The data indicated large tumors have more blood vessels delivering oxygen to the tumor in order to sustain the growth, and hence we see an increased StO_2_ value between the two days. The changes in StO_2_ 24 h (D12) and 72 h (D14) after therapy were also correlated with various treatment growth parameters. The D10–D14 StO_2_ had overall good correlation with alpha, beta, AUC_Early_, 2×, 5×, and 10× (r^2^ = 0.7, r^2^ = -0.53, r^2^ = 0.65, r^2^ = 0.74, and r^2^ = 0.82 with respective p-values of 0.0003, 0.032, 0.045, 0.019, and 0.004). D10–D12 had moderate correlation with 2×, 5×, and AUC_Early_ [r^2^ = 0.47 (p = 0.269), r^2^ = 0.45 (p = 0.0574), and r^2^ = 0.56 (p = 0.0231), respectively] but not with other parameters. A good statistical correlation was probably not observed for StO_2_ D10–D12 value because initiation of therapy had only minimal impact at 24 h posttreatment. A scatter bubble plot of changes in StO_2_ values between D10 and D14 vs. tumor growth rate β (day^-1^) is shown in **Figure 7**. The changes in StO_2_ from D7 to D10 are indicated by pseudo color where a decrease in oxygenation is represented by red, while an increase in oxygenation on D10 is represented by blue. The size of the bubbles is representative of the pretreatment volume of the tumors on D10. The label “0” indicates tumors in the control group, while 1 indicates the treated group. The treated and the control groups formed distinct clusters with minimal overlap, although the cluster did not show any significant trends with respect to pretreatment tumor volume. Within the treated cluster in **Figure 7**, we clearly notice that if tumors became hypoxic between D7 and D10 (purple-red color), they had a significantly lower change in oxygenation between D10 and D14. Tumors that became more oxygenated from D7 to D10 (dark purple-blue color) had a greater change in D10–D14 StO_2_ values posttreatment and also lower growth rate. The observation supports previous studies that showed that cabozantinib is efficacious in highly angiogenic tumors, as these tumors have a high expression of VEGFR, a target receptor suppressed by cabozantinib (50, 51). Reliable trends on pretreatment tumor volume and change in StO_2_ between D7 and D10 are not observed, as also indicated in the correlation matrix (r^2^ = -0.504 and p = 0.058).

**Figure 8:**
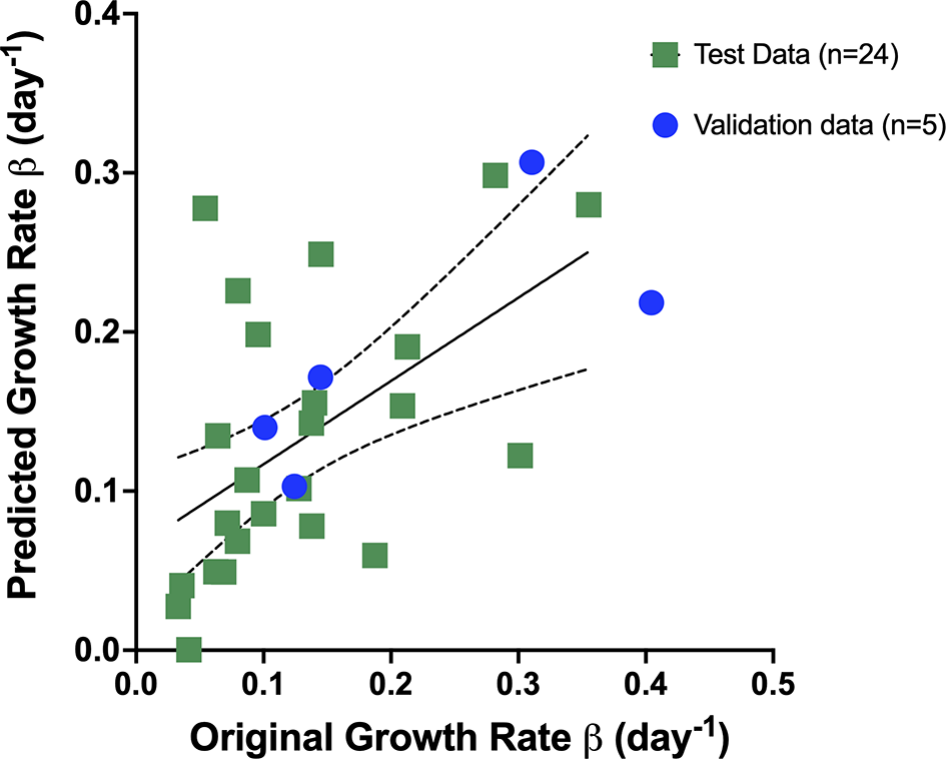
Representative data from the k-fold cross-validation of the multivariate linear regression model given by the Equation: *Growth rate β* = *b*_0_+ *b*_1_∗ *pretreatment* *volume* *b*_2_ ∗ *StO*_2_(*D*7−*D*10) +*b*_3_∗*StO*_2_(*D*10−*D*14) . The training data set is represented by green squares (n = 24), the representative regression line in black, and the validation data set is represented by blue circles (n = 5).

We utilized the relevant parameters with good correlation coefficient with Gompertz fit parameter β value, namely, pretreatment tumor volume and change in StO_2_ pretreatment (D7–D10) and immediately following treatment initiation (D10–D14) to develop our multivariate linear regression model, where Growth rate *β* = *b*_0_+ *b*_1_∗ *pretreatment* *volume* *b*_2_ ∗ *StO*_2_(*D*7−*D*10) +*b*_3_∗*StO*_2_(*D*10−*D*14)  and StO_2_ (D7–D10) and StO_2_ (D10–D14) represent the change in StO_2_ between Days 7 and 10 and immediately following treatment initiation on Days 10–14, respectively. The AUC_Early_ parameter had a better correlation with Gompertz growth rate and StO_2_ parameters compared to the “pretreatment” tumor volume. However, obtaining AUC_Early_ clinically with several days prior to initiation of treatment is not possible, as treatment is given immediately post-tumor detection. Hence, immediately prior to treatment tumor volume on D10 was considered in the prediction model. To enhance the performance of the model, mice that were treated with different cabozantinib doses were added to the data. Gompertz β parameter was the dependent (output) variable. A total of 29 mice were used to train and validate the model, with three predictors (independent variable) allowing ~10 cases per predictor in the multivariate regression model, satisfying the minimum required observations per predictor to avoid overfitting (52, 53). A forward selection method without cross-validation was used to determine the three best predictors based on the R^2^, adjusted R^2^ value, and the coefficient for regression p-value <0.05. Multicollinearity was assessed using Variance Inflation Factor with a cutoff of 3. The process yielded that a linear combination of pretreatment tumor volume, D7–D10, and D10–D14 had the highest R^2^ value. A table of R^2^ values for different combinations of predictors (3 predictors or less) is provided in the Supplementary Material (**Table S9**). The k-fold cross-validation method (k = 5 samples) and the random subsampling method (80% training data and 20% validation data; 100 repeats) yielded similar regression coefficients (**Table 1**). **Figure 8** shows the representative data from the k-fold cross-validation on the training data set (green square data points), the representative regression line, and the validation data set (blue round data points). The low R^2^ values with low p-values in this regression analysis indicate that the data are noisy; however, there is a significant trend between growth rate and the parameters pretreatment volume and StO_2_ changes pretreatment and posttreatment.

**Table 1:** Summary of parameter estimates for *b*_0_, *b*_1_, *b*_2_, and *b*_3_ obtained from multiple linear regression along with corresponding R^2^ and p-values.

	Multiple linear regressionparameter estimates (standard error)					
	*b*_0_ Intercept	*b*_1_ Pretreatment volume	*b*_2_ StO_2_ D7–D10	*b*_3_ StO_2_ D10–D14	R^2^	p-value
No-treatment and high-dose	0.27 (0.076)	-0.002 (0.0013)	-0.0074 (0.0026)	-0.0042 (0.0018)	0.58	0.02
All groups	0.16 (0.056)	-0.00033 (0.00086)	-0.0052 (0.0021)	-0.0041 (0.0014)	0.34	0.014
k-fold cross-validation	0.1535 (0.0216)	-0.0002 (0.003)	-0.0052 (0.006)	-0.0042 (0.002)	0.37	0.011
Random subsampling cross-validation	0.1580 (0.039)	-0.0003 (0.0012)	-0.0053 (0.0001)	-0.0042 (0.0001)	0.37	0.0065

Size groups used were n = 15 and 29 for no-treatment and cabozantinib and all mice respectively. For the k-fold cross-validation, k = 5 was used to represent the number of groups all mice were split into. Lastly, random subsampling cross-validation (N = 100 repeats) consisted of 80% training data and 20% validation data.

**Figure 9:**
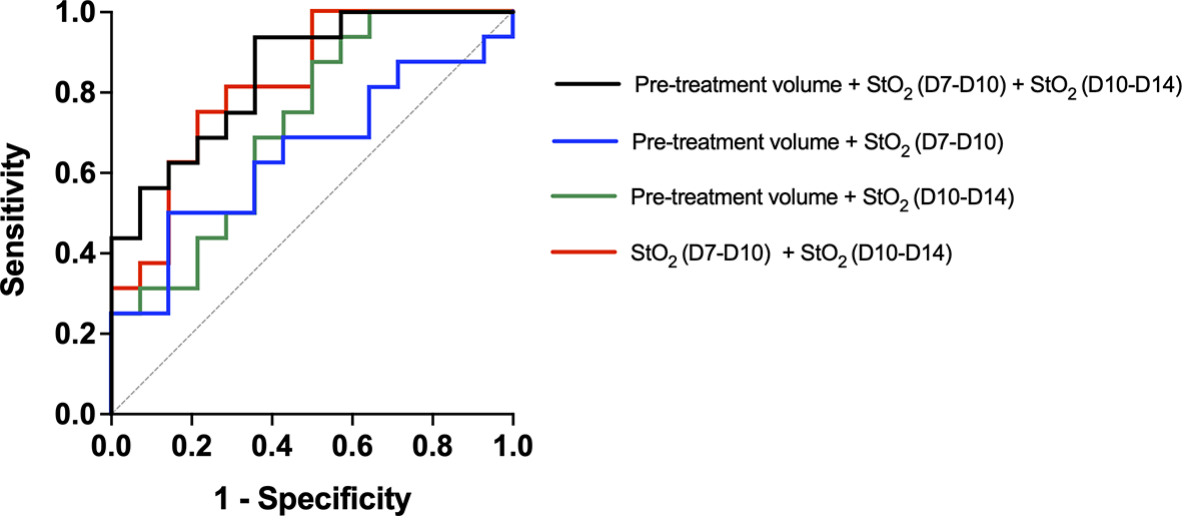
Receiver operating characteristic curve showcasing the predictive capability of various parameters used in the multiple logistic regression analysis. The model created with Pretreatment volume and change in StO_2_ between D7–D10 and D10–D14 had the highest AUC. The line of identity is shown as a gray dotted line.

We used multiple logistic regression analysis, a popular and widely used analysis similar to linear regression analysis, to evaluate the performance of the three parameters, namely, pretreatment tumor volume and StO_2_ D7–D10 and D10–D14 in predicting the treatment response. The outcome in logistic regression analysis is dichotomous, i.e., responders (1) or non-responders (0). As mice with various treatment doses are included in the analysis, we allocated a value of “1” for tumors that had a Gompertz growth rate lower than the 25th percentile of the no-treatment group, i.e., this group was identified as responders and other tumors were non-responders. Goodness-of-fit of the logistic regression model was performed with the Hosmer–Lemeshow (HL) test where the hypothesis is that predictions agree well with observed outcomes and a p-value greater than 0.05 indicated good agreement. In all of the logistic regression analyses shown in **Figure 9**, p-values greater than 0.05 were obtained. All of the 29 mice were included in the analysis, and a receiver operating characteristic (ROC) curve was utilized to assess the predictive efficacy of the three parameters. The area under the ROC curve (AUC) is a measure of how well the fit model correctly classified non-responding and responding tumors. The classification cutoff threshold point was determined to be 0.8 by maximizing the Youden function, which is the difference between true positive rate and false-positive rate over all possible cutoff values. As expected, the AUC of the ROC curves for a single variable did not have a high AUC (pretreatment volume, StO_2_ D7–D10, or StO_2_ D10–14), although the change in StO_2_ values had better predictive capability than pretreatment tumor volume. **Figure 9** showcases the ROC curves indicative of the performance of combination parameters (either two or all three parameters). The combination of pretreatment conditions (tumor volume and D7–D10 change in StO_2_, **Figure 9**, blue line) had the least predictive capability with AUC 0.580 (standard error of 0.108, 95% confidence interval 0.370–0.791, and p-value of 0.454). Combining pretreatment tumor volume with change in StO_2_ posttreatment D10–D14 had an AUC of 0.72 (standard error of 0.095, 95% confidence interval of 0.533–0.905, and p-value of 0.042). The combination of StO_2_ changes pretherapy and post-therapy (D7–D10 and D10–D14) had an AUC of 0.82 (standard error of 0.077, 95% confidence interval of 0.670–0.973, and p-value of 0.0028). The combination of all three parameters had the best AUC of 0.85 (standard error of 0.06903, 95% confidence interval of 0.713–0.984, p = 0.0012), however, was not statistically different from the D7–D10 to D10–D14 ROC curve. Although pretreatment tumor volume has previously been shown clinically and preclinically as a predictor of response, in this data set, the parameter is insignificant probably due to the limited range of tumor volumes used in the study. Furthermore, the model built with the three predictors had a 100% positive predictive power, 58.33% negative predictive power, 100% specificity, and 30% sensitivity. The high positive predictive power indicates the probability that people with a positive prediction result indeed do have responded to the treatment and have a lower growth rate. The model can correctly identify from among the sample which tumor might or might not have responded to the treatment. The low sensitivity indicates that the model can identify solely from among tumors that are known to have a good response to the treatment (i.e., identifying true positives). However, our model will err on the side of caution and may not identify a responsive tumor if the data are perhaps borderline. The high specificity would indicate that our model can correctly identify tumors that are not responsive to treatment.

## Conclusions and Future Work

In the current study, we have demonstrated the effectiveness of US-PAI to monitor vascular biomarkers to detect changes in tumor responsiveness to oral TKI therapy early in the treatment regimen. US-PAI does not require any exogenous contrast agents, can be performed by trained technicians, and in real time provide data feedback on the tumor structure and vascular elements. By acquiring both pretreatment and posttreatment data, we characterized and distinguished between treated and non-treated tumors. We then developed a prediction model for tumor growth rate based on the pretreatment tumor volume, pretreatment change in StO_2_, and posttreatment change in StO_2_ parameters. Personalizing a patient’s treatment regimen and gauging will be invaluable in the clinical setting, and US-PAI has tremendous potential to noninvasively track daily changes in hemodynamics to predict treatment response.

PAI offers unprecedented 3D information on tumor vascular function, especially information on StO_2_, that can aid in gauging heterogeneity in the TME (54, 55). However, the StO_2_ estimation is dependent on the photoacoustic signal strength that is in turn wavelength-dependent on the fluence at a particular depth (56–58). The estimated StO_2_ values can become erroneous for larger tumors due to significant differences in the fluence at deeper tissues due to wavelength-dependent scattering and absorption of light by tissue. These values can also be further influenced by the light delivery system, i.e., focused beam vs. large beam diameter can lead to different fluencies at various depths (59). In the current setup, the laser light was focused at around 10 mm from the transducer surface, and we clearly showcase that StO_2_ measurements in water (no scattering) are influenced by the position of the tube filled with oxygenated hemoglobin. For shallower depths where sufficient light is not reaching the tube, very low StO_2_ values were recorded for tube filled with oxygenated hemoglobin (**Figure S5**). When the tube is placed in a scattering medium, there was no statistically significant difference in the StO_2_ measured from the tube at various depths between 5 and 18 mm (**Figure S5**). In our experiments, we ensured that the placement of the tumors was between 8 and 10 mm from the transducer to avoid situations of the tumor ROI being placed beyond the light focus.

Recent simulations by Yoon et al. (60), Hochuli et al. (58, 61), and Perekatova et al. (62) demonstrate that wavelengths around 680–1,000-nm range can be used for measuring StO_2_ values at various depths; however, further experiments need to be conducted prior to deciding the optimal wavelength combination for a particular application. Here, we used the dual-wavelength imaging at 750 and 850 nm, as they straddle around the isosbestic point of oxygenated and deoxygenated hemoglobin optical absorption curves, and these settings are currently unchangeable within the “Oxy-Hemo” imaging mode of the Vevo LAZR-X system. While the differences in wavelength-dependent laser energy are compensated within the system, depth-dependent fluence compensation is not available. Several groups utilized the dual-wavelength “Oxy-Hemo” mode for applications ranging from monitoring treatment response (37, 41) to measuring ischemia–reperfusion in humans (63) and oxygen gradients in the retina (64). Rich and Seshadri (65) have also demonstrated good correlation of photoacoustic StO_2_ values measured with Vevo LAZR-X imaging system with BOLD-MRI. Light-emitting diode-based PAI systems have also utilized the wavelength combination of 750 and 850 nm to measure StO_2_ changes in mice and humans (66). Kim et al. (39) performed a study analyzing StO_2_ values obtained from various combinations of wavelengths and their predictive capability for discerning benign and malignant thyroid lesions. StO_2_ measurements calculated using five wavelengths (700, 756, 796, 866, and 900 nm) had higher specificity than utilizing a combination of 2, 3, or 4 wavelengths, respectively. It is to be noted that 3D images of the tumor were not acquired in the Kim et al. study but rather 2D cross-sections were utilized to differentiate benign and malignant lesions. While the dual-wavelength 750 and 850 nm combination might not yield accurate StO_2_ measurements compared to multiple wavelengths, methods using more than two wavelengths to discern StO_2_ will also significantly increase the scan time. Indeed, the trade-off between StO_2_ measurement accuracy and scan speed to obtain a 3D image is yet to be studied in detail and could be resolved with the availability of lasers with a higher pulse repetition frequency.

The accuracy of our StO_2_ measurements can be improved with depth-dependent changes in fluence estimated using Monte Carlo simulations (67, 68), by obtaining wavelength-dependent optical attenuation measured using photoacoustic spectra of 25-µm-thick black film (69), using ultrasonic tagging of light (70), utilizing radiofrequency photoacoustic spectra (71), or using signal-to-noise ratio-regularized local fluence correction (72). More recently, deep learning methodologies for spectral unmixing of photoacoustic signals have also been proposed for accurate StO_2_ measurements (73, 74). Hochuli et al. also demonstrated that spectral coloring introduces significant inaccuracies in the StO_2_ estimation. High density of blood vessels in the top layers of the tumor can also impact StO_2_ measurements in deeper tissues due to significant absorption of light by the blood vessels in the top layers of the tumor. Methods to compensate for fluence in such heterogeneous environments and large tumors are not straightforward and require further investigation. This can be further exacerbated in cases such as skin tumors where the presence of melanin can also impact StO_2_ measurements, as recently demonstrated by Mantri and Jokerst (75). The above-stated caveats for accurately estimating StO_2_ including the number of wavelengths required for spectal unmixing, depth-dependent fluence compensation, and heterogeneous TME leading to heterogeneous optical absorption within the tumor need to be addressed prior to clinical translation. Nevertheless, the prediction model presented in this article was developed with tumor volumes in the range of ~45–85 mm^3^ and is a promising step toward utilizing noninvasive label-free imaging biomarkers to predict treatment response. Such methods can be integrated into preclinical cancer research to comprehensively evaluate the variations in therapy response.

Future work will include incorporating orthotopic models with treatment-resistant pancreatic cancer cell lines and studying other forms of TKI therapies at effective and suboptimal therapy doses to develop a robust prediction model. Our work demonstrated the possibility to monitor minute vascular changes in oxygenation *via* oral administration of the TKI while tracking volume changes through our treatment regimen. We believe that these changes may become even more pronounced through intravenous delivery of the therapies, as oral delivery may not produce as drastic volumetric reductions as anticipated and seen in previous studies (76). Furthermore, recent developments in clinical translation of PAI (77–79) including portable real-time LED-based PAI systems for cancer applications (80) and endoscopic PAI systems (81–83) show promise toward employing photoacoustic monitoring of tumor response to treatment not only for pancreatic tumors but also for other solid tumors undergoing neoadjuvant treatment. Overall, given the exponential rise in the technical advances and biological applications of PAI over the past decade, the results presented in this study further support its utility as a useful tool to monitor cancer treatment response, especially in imaging response of suboptimal therapies.

## Data Availability Statement

The raw data supporting the conclusions of this article will be made available by the authors without undue reservation.

## Ethics Statement

The animal study was reviewed and approved by Tufts University.

## Author Contributions

The authors confirm contribution to the paper as follows: Study conception and design: SM. Experimentation and data collection: AC, AS, DS, BL, DW, and MX. Analysis and interpretation of results: AC, AS, and SM. Draft article preparation: AC, AS, and SM. Funding: SM. All authors reviewed the results and approved the final version of the article.

## Funding

The authors would like to acknowledge funds from NIH S10 0D026844 and R21 CA263694 grants, Tufts University School of Engineering, and Tufts University Data Intensive Studies Center.

## Conflict of Interest

The authors declare that the research was conducted in the absence of any commercial or financial relationships that could be construed as a potential conflict of interest.

## Publisher’s Note

All claims expressed in this article are solely those of the authors and do not necessarily represent those of their affiliated organizations, or those of the publisher, the editors and the reviewers. Any product that may be evaluated in this article, or claim that may be made by its manufacturer, is not guaranteed or endorsed by the publisher.
