# Supplementary material for: 3D Ultrasound-Guided Photoacoustic Imaging to Monitor the Effects of Suboptimal Tyrosine Kinase Inhibitor Therapy in Pancreatic Tumors
Source: Front Oncol. 2022 Jul 7;12:915319. doi: 10.3389/fonc.2022.915319 (PMC9300843; doi:10.3389/fonc.2022.915319)
Supplement: Supplementary file 1 [file DataSheet_1.docx]

Supplementary Material

**Supplementary Figure 1.**

| 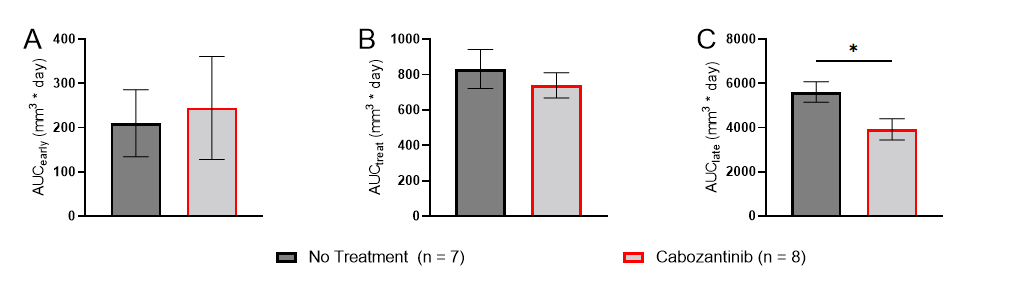 |
| --- |
| **Figure S1.** Box plot comparing the (A) AUC_Early_ (B) AUC_Treat_ and (C) AUC_Late_ for each group. Error bars indicate S.E.M. A two-way, unpaired t-test provided p-value of 0.026 indicating statistical difference between the two groups for AUC_Late_ term. *= p-value < .05 |

**Supplementary Figure 2.**

| 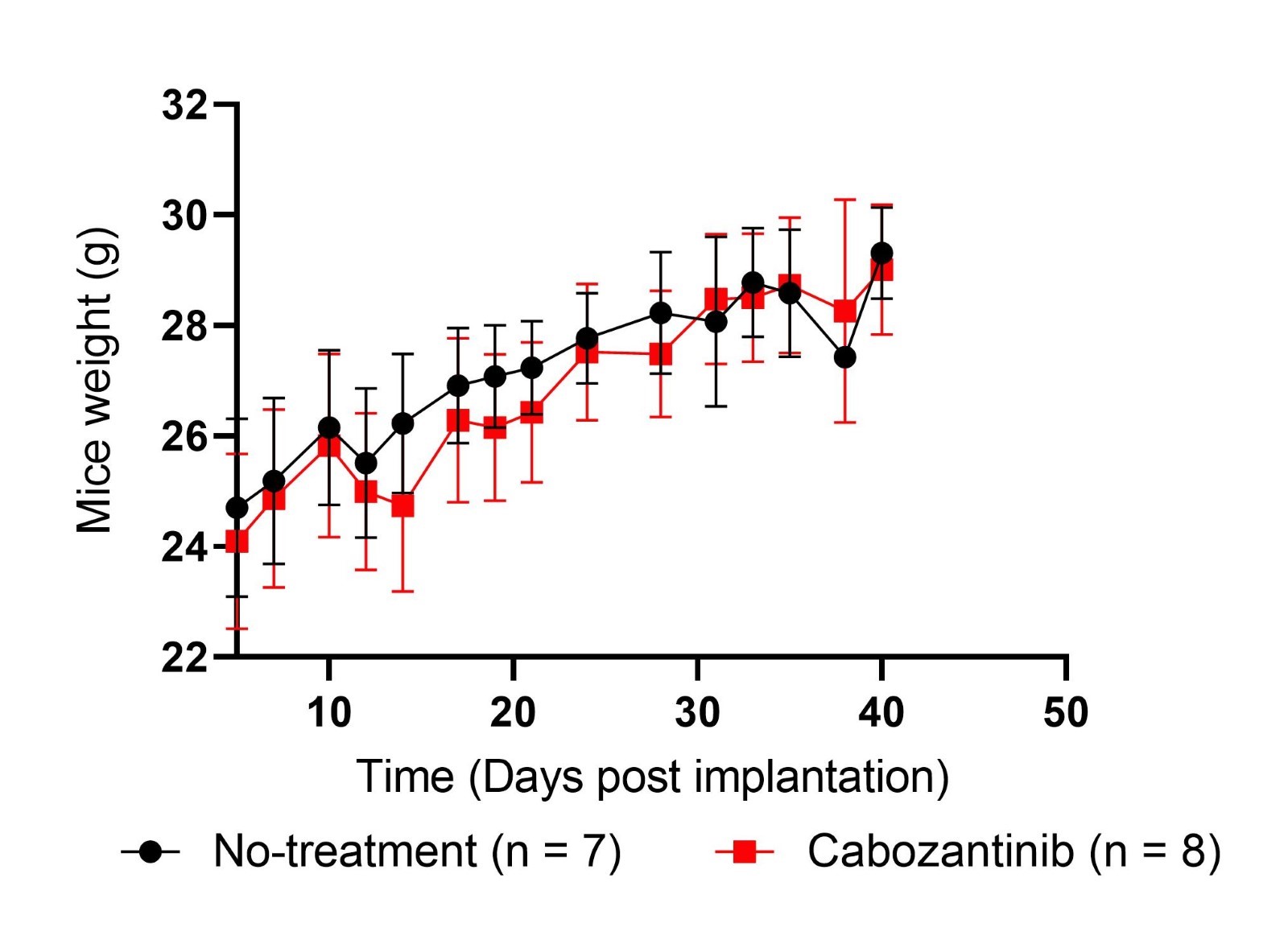 |
| --- |
| **Figure S2.** Weight of mice during the course of the study. Error bars represent S.E.M. Performing multiple, two-way, unpaired t-tests reveals no statistical significance between the two groups. |

**Supplementary Figure 3.**

| 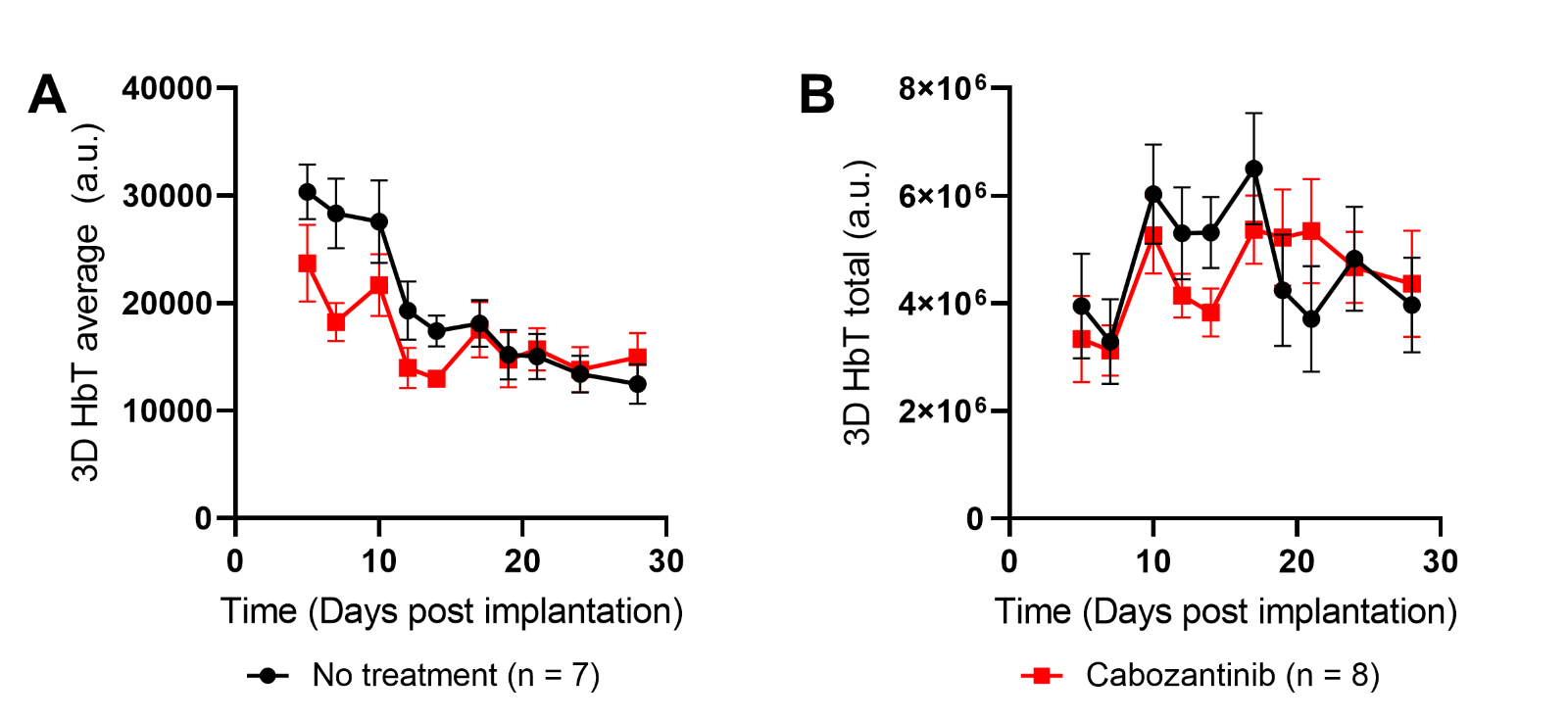 |
| --- |
| **Figure S3:** Mean of (A) average hemoglobin concentration and (B) total hemoglobin concentration values over first 30 days post implantation are shown for both groups. Error bars represent S.E.M. No statistically significant difference was observed between the groups. |

**Supplementary Figure 4**

| 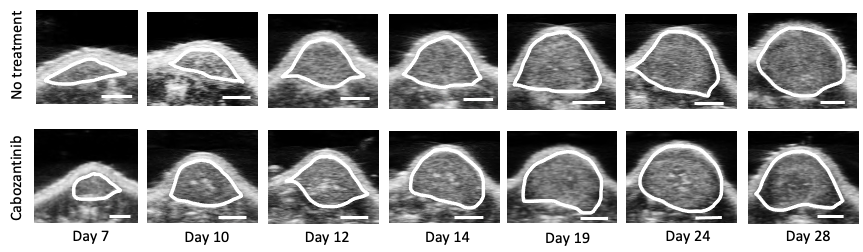 |
| --- |
| **Figure S4:** 2D Ultrasound images of the control (no-treatment) and cabozantinib treated tumors on Day 7, 10, 12, 14, 19, 24, and 28 post-implantation. The scale bar represents 2 mm. White ROI represents the tumor region. |

**Supplementary Figure 5.**

| 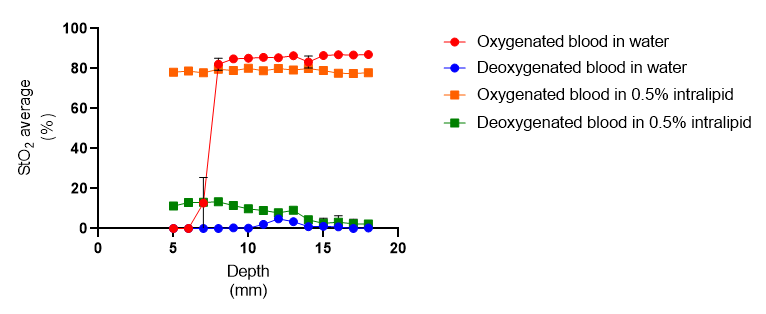 |
| --- |
| **Figure S5.** Plot of StO_2_ average vs imaging depth of a 100% oxygenated and deoxygenated hemoglobin filled tube placed in a tank filled with deionized water or 0.5% intralipid scattering medium. The error bars shown represent standard error mean at each depth. |

# Supplementary Tables

**Supplementary Table 1.**

**Table S1:** Comparison of region of interest (ROI) values from the cabozantinib group against the not-treated groups at various time points using multiple unpaired t tests.

| **Unpaired t test** | **Difference in the mean** | **Significant?**  **P < 0.05** |
| --- | --- | --- |
| Day 5 | -11.4 | No |
| Day 7 | -7.19 | No |
| Day 10 | -7.71 | No |
| Day 12 | -1.96 | No |
| Day 14 | 7.69 | No |
| Day 17 | 8.79 | No |
| Day 19 | 50.4 | No |
| Day 21 | 54.4 | Yes |
| Day 24 | 57.3 | Yes |
| Day 28 | 74.9 | Yes |
| Day 31 | 124 | Yes |
| Day 33 | 83.2 | Yes |
| Day 35 | 130 | Yes |
| Day 38 | 32.8 | No |
| Day 40 | 76.5 | Yes |

**Supplementary Table 2.**

**Table S2:** Comparison of caliper measurements of the tumors from the cabozantinib group against the no-treatment group at various time points using multiple unpaired t tests.

| **Unpaired t test** | **Difference in the mean** | **Significant?**  **P < 0.05** |
| --- | --- | --- |
| Day 5 | -6.281 | No |
| Day 7 | 0.005158 | No |
| Day 10 | 0.6628 | No |
| Day 12 | -15.12 | No |
| Day 14 | -0.1922 | No |
| Day 17 | 24.75 | No |
| Day 19 | 38.59 | No |
| Day 21 | 54.34 | Yes |
| Day 24 | 64.78 | Yes |
| Day 28 | 80.45 | Yes |
| Day 31 | 90.39 | Yes |
| Day 33 | 108.5 | Yes |
| Day 35 | 75.50 | Yes |
| Day 38 | 57.75 | No |
| Day 40 | 108.3 | Yes |

**Supplementary Table 3.**

**Table S3:** Variation in StO_2_ average, total, and HbT average and total values for three images taken in repeatability experiment for n = 3 mice. The average variability between 41 co-registered frames of the three images is reported for each mouse. The average over all mice is taken to report the combined variability for each parameter.

| **Mouse #** | **StO_2_ avg**  **(%)** | **StO_2_ total**  **(%)** | **HbT avg**  **(a.u.)** | **HbT total**  **(a.u.)** |
| --- | --- | --- | --- | --- |
| 1 | 5.523 ± 1.780 | 3.434 ± 0.912 | 2091 ± 424.1 | 3.35 x 10^7^ ± 1.27 x 10^7^ |
| 2 | 5.865 ± 1.572 | 11.96 ± 3.846 | 3850 ± 836.2 | 4.36 x 10^7^ ± 1.30 x 10^7^ |
| 3 | 2.990 ± 2.000 | 6.830 ± 2.060 | 3078 ± 2059 | 4.17 x 10^7^ ± 3.32 x 10^7^ |
| **Mean** | **4.793 ± 1.784** | **7.408 ± 2.275** | **3006 ± 1106** | **3.96 x 10^7^ ± 1.96 x 10^7^** |

**Supplementary Table 4**

**Table S4:** Comparison of average oxygen saturation (StO_2_) values from the cabozantinib group against the not treated groups at various time points using multiple unpaired t tests.

| **Unpaired t test** | **Difference in the mean** | **Significant?**  **P < 0.05** |
| --- | --- | --- |
| Day 5 | -3.353 | No |
| Day 7 | -7.052 | No |
| Day 10 | -4.522 | No |
| Day 12 | 5.398 | No |
| Day 14 | 10.39 | Yes |
| Day 17 | -6.313 | No |
| Day 19 | -2.016 | No |
| Day 21 | -2.548 | No |
| Day 24 | -6.910 | No |
| Day 28 | -9.668 | Yes |
| Day 31 | -3.353 | No |
| Day 33 | -4.698 | No |
| Day 35 | -2.116 | No |
| Day 38 | 6.602 | No |
| Day 40 | -4.932 | No |

**Supplementary Table 5.**

**Table S5:** Comparison of total oxygen saturation (StO_2_) values from the cabozantinib group against the not treated groups at various time points using multiple unpaired t tests.

| **Unpaired t test** | **Difference in the mean** | **Significant?**  **P < 0.05** |
| --- | --- | --- |
| Day 5 | -1.117 | No |
| Day 7 | 8.566 | No |
| Day 10 | 3.905 | No |
| Day 12 | 13.51 | Yes |
| Day 14 | 17.44 | Yes |
| Day 17 | 3.054 | No |
| Day 19 | 1.746 | No |
| Day 21 | -0.5352 | No |
| Day 24 | -3.654 | No |
| Day 28 | -14.87 | Yes |
| Day 31 | -12.45 | Yes |
| Day 33 | -14.99 | No |
| Day 35 | -8.615 | No |
| Day 38 | -10.20 | No |
| Day 40 | -6.763 | No |

**Supplementary Table 6.**

**Table S6:** Comparison of 3D average hemoglobin concentration values (HbT) from the cabozantinib group against the no treatment group for the first 28 days, through treatment. These differences were calculated through multiple two-way, unpaired t tests.

| **Unpaired t test** | **Difference in the mean** | **Significant?**  **P < 0.05** |
| --- | --- | --- |
| Day 5 | 6639 | No |
| Day 7 | 10113 | Yes |
| Day 10 | 5903 | No |
| Day 12 | 5320 | No |
| Day 14 | 4450 | No |
| Day 17 | 560.7 | No |
| Day 19 | 468.7 | No |
| Day 21 | -667.2 | No |
| Day 24 | -396.7 | No |
| Day 28 | -2513 | No |

**Supplementary Table 7.**

**Table S7:** Comparison of 3D total hemoglobin concentration values (HbT) from the Cabozantinib group against the no treatment group for the first 28 days, through treatment. These differences were calculated through multiple two-way, unpaired t tests.

| **Unpaired t test** | **Difference in the mean** | **Significant?**  **P < 0.05** |
| --- | --- | --- |
| Day 5 | 613821 | No |
| Day 7 | 163481 | No |
| Day 10 | 765549 | No |
| Day 12 | 1159755 | No |
| Day 14 | 1483898 | No |
| Day 17 | 1130847 | No |
| Day 19 | -982680 | No |
| Day 21 | -1630774 | No |
| Day 24 | 158924 | No |
| Day 28 | -390706 | No |

**Supplementary Table 8.**

**Table S8:** Spearman correlation coefficients of StO_2_ total Days 10, 12, 14, 17 correlated with Gompertz function parameters (α, β, and κ), time taken for the tumors to reach twice (2X), five times (5X) and ten times (10X) their pre-treatment volume, Day 10 volume, and area under the pre-treatment volume (AUC_Early_), treatment volume (AUC_Treat_), and post-treatment volume (AUC_Late_) growth curves.

|  | **α** | **β** | **κ** | **AUC**  **Early** | **AUC**  **Treat** | **AUC**  **Late** | **Vol Day 10** | **2X** | **5X** | **10X** |
| --- | --- | --- | --- | --- | --- | --- | --- | --- | --- | --- |
| **StO_2_ total**  **Day10** | 0.171 | -0.003 | 0.106 | 0.485 | -0.021 | -0.027 | 0.159 | 0.153 | 0.237 | 0.427 |
| **StO_2_ total**  **Day12** | -0.377 | 0.418 | 0.438 | -0.168 | -0.291 | 0.065 | -0.259 | -0.344 | -0.277 | -0.179 |
| **StO_2_ total**  **Day14** | -0.127 | 0.315 | 0.268 | 0.061 | 0.072 | 0.129 | 0.029 | -0.500 | -0.427 | -0.306 |

**Supplementary Table 9.**

**Table S9:** A forward selection method without cross validation was used to determine the three best predictors of β based on the R-squared, adjusted R-squared value and statistical significance of the coefficient for regression p-value <0.05. This table displays these values for different combinations of predictors (3 predictors or less).

| **Parameters** | **R^2^ (adjusted R^2^ value)** | **p-value** |
| --- | --- | --- |
| β ~ Intercept + D7 + D14 | 0.50 (0.42) | 0.016 |
| β ~ Intercept + (D10 - D14) | 0.24 (0.18) | 0.065 |
| β ~ Intercept + D7+ (D10 - D14) | 0.29 (0.17) | 0.130 |
| β ~ Intercept + (D7-D10) + (D10 - D14) | 0.48 (0.39) | 0.020 |
| β ~ Intercept + Pretreatment volume + (D7-D10) + (D10 - D14) | 0.58 (0.46) | 0.020 |
| β ~ Intercept + Pre-treatment volume + (D10 - D14) | 0.27 (0.15) | 0.154 |
